# Assisted reproductive technology in Japan: A summary report for 2019 by the Ethics Committee of the Japan Society of Obstetrics and Gynecology

**DOI:** 10.1002/rmb2.12434

**Published:** 2021-12-14

**Authors:** Yukiko Katagiri, Seung Chik Jwa, Akira Kuwahara, Takeshi Iwasa, Masanori Ono, Keiichi Kato, Hiroshi Kishi, Yoshimitsu Kuwabara, Miyuki Harada, Toshio Hamatani, Yutaka Osuga

**Affiliations:** ^1^ Department of Obstetrics and Gynecology Faculty of Medicine Toho University Tokyo Japan; ^2^ Department of Obstetrics and Gynecology Saitama Medical University Saitama Japan; ^3^ Department of Obstetrics and Gynecology Graduate School of Biomedical Sciences Tokushima University Tokushima Japan; ^4^ Department of Obstetrics and Gynecology Tokyo Medical University Tokyo Japan; ^5^ Kato Ladies Clinic Tokyo Japan; ^6^ Department of Obstetrics and Gynecology The Jikei University School of Medicine Tokyo Japan; ^7^ Department of Obstetrics and Gynecology Nippon Medical School Tokyo Japan; ^8^ Department of Obstetrics and Gynecology Graduate School of Medicine The University of Tokyo Tokyo Japan; ^9^ Department of Obstetrics and Gynecology School of Medicine Keio University Tokyo Japan

**Keywords:** assisted reproductive technology, cryopreservation, intracytoplasmic sperm injections, Japan, registries

## Abstract

**Purpose:**

The Japan Society of Obstetrics and Gynecology records online annual cycle‐based information for assisted reproductive technology (ART). This report presents the characteristics and treatment outcomes of ART cycles registered during 2019.

**Methods:**

The Japanese ART registry includes cycle‐specific information from 619 participating facilities, including treatment and pregnancy outcomes. Descriptive analyses were conducted for cycles registered during 2019.

**Results:**

In 2019, 458 101 treatment cycles and 60 598 neonates were reported, both of which increased from 2018. The number of fresh cycles, including in vitro fertilization and intracytoplasmic sperm injection, decreased, while frozen‐thawed embryo transfer (ET) cycles increased. The mean maternal age was 37.9 years (standard deviation ± 4.7). Of 239 348 oocyte retrievals, 123 690 (51.7%) involved freeze‐all‐embryos cycles; fresh ET was performed in 41 831 cycles (a decreasing trend since 2015). In 2019, there were 211 597 frozen‐thawed ET cycles, resulting in 74 882 pregnancies and 54 168 neonates born. Single ET was performed in 82.6% of fresh transfers and 85.1% of frozen‐thawed cycles, with singleton live birth rates of 97.3% for both.

**Conclusions:**

The number of fresh cycles decreased but frozen cycles increased in 2019. Single ET was performed in >80% of cases, and the proportion of babies born from frozen‐thawed ET increased.

## Graphical abstract

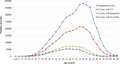

## 1 INTRODUCTION

Since the first baby was born by in vitro fertilization (IVF) in 1983 in Japan, the number of assisted reproductive technology (ART) cycles has increased each year. According to the latest preliminary report from the International Committee Monitoring Assisted Reproductive Technologies in 2016, Japan was the second‐largest national user of ART globally, based on the total annual number of treatment cycles implemented.^1^

Because the fertility rate in Japan has been decreasing steadily since the 1980s,^2^ the contribution of ART to the overall birth rate in Japan is increasing. To evaluate the effectiveness and safety of ART and to understand the latest situations requiring the implementation of ART, it is essential to monitor national trends of use and outcomes of implemented ART treatments. The Japan Society of Obstetrics and Gynecology (JSOG) started an ART registry system in 1986 and launched an online registration system in 2007. From this, the cycle‐specific information of all ART treatment cycles performed in ART facilities in Japan can be assembled. The aim of this report was to describe the characteristics and treatment outcomes of registered ART cycles during 2019 in comparison with previous years.^3^

## 2 MATERIALS AND METHODS

Since 2007, the JSOG has obtained cycle‐specific information from all ART treatment cycles of participating ART facilities, including clinics and hospitals. This information includes patient characteristics such as infertility diagnosis, information on the specific ART treatment (eg, IVF and intracytoplasmic sperm injection [ICSI]), and pregnancy and obstetric outcomes. Detailed information collected by this registry has been reported previously.^4^ For ART cycles implemented between January 1 and December 31, 2019, the JSOG requested that online registration, including the input of all relevant information, be carried out by the end of November 2020.

Using the registry data from 2019, we performed a descriptive analysis to investigate the characteristics and treatment outcomes of registered cycles. First, the numbers of registered cycles, oocyte retrievals, embryo transfer (ET) cycles, freeze‐all‐embryo/oocyte cycle (henceforth “freeze‐all”) pregnancies, and neonates were compared with those in previous years for IVF, ICSI, and frozen‐thawed embryo transfer (FET) cycles. Second, the characteristics of registered cycles and pregnancy outcomes were described for fresh and FET cycles. Fresh cycles were stratified according to fertilization methods, including IVF, ICSI, and gamete intrafallopian transfer (GIFT), and included cycles with oocyte freezing based on medical indications. Treatment outcomes included clinical pregnancy (defined as confirmation of a gestational sac in utero), miscarriage (defined as spontaneous or unplanned loss of a fetus from the uterus before 22 weeks of gestation), live birth (defined as the delivery of at least one live neonate after 22 weeks of gestation), and multiple pregnancy rates. Pregnancy outcomes included ectopic pregnancy, heterotopic pregnancy, artificially induced abortion, stillbirth, and fetal reduction. Third, the treatment outcomes of pregnancy, live birth, miscarriage, and multiple pregnancy rates were analyzed according to patient age. Last, the treatment outcomes for FET cycles using frozen‐thawed oocytes were investigated.

## 3 RESULTS

In Japan, there were 624 registered ART facilities in 2019, of which 619 participated in the ART registration system. A total of 595 registered facilities implemented some form of ART treatment in 2019, while 24 did not. Trends in the numbers of registered cycles, oocyte retrievals, pregnancies, and neonates born by IVF, ICSI, and FET cycles since 1985 are shown in Table 1. In 2019, 458 101 cycles were registered and 60 598 neonate births were recorded. The total number of registered cycles for both IVF and ICSI had decreased from 2018 to 2019. The numbers of freeze‐all IVF and ICSI cycles increased in 2019, and the number of neonates born was 2974 for IVF‐ET cycles and 3433 for ICSI cycles, both of which were decreased from the previous year. In contrast, the number of FET cycles has increased continuously since the late 1980s; in 2019, there were 215 203 cycles registered (a 5.8% increase from 2018), resulting in 74 911 pregnancies and 54 188 neonates born.

**TABLE 1:** Trends in numbers of registered cycles, egg retrievals, pregnancy, and neonate births categorized by IVF, ICSI, and frozen‐thawed embryo transfer cycles in Japan (1985–2019)

Year	Fresh cycles												FET cycles^c^			
	IVF^a^						ICSI^b^									
	No. of registered cycles	No. of egg retrievals	No. of freeze‐all cycles	No. of ET cycles	No. of cycles with pregnancy	No. of neonates	No. of registered cycles	No. of egg retrievals	No. of freeze‐all cycles	No. of ET cycles	No. of cycles with pregnancy	No. of neonates	No. of registered cycles	No. of ET cycles	No. of cycles with pregnancy	No. of neonates
1985	1195	1195		862	64	27										
1986	752	752		556	56	16										
1987	1503	1503		1070	135	54										
1988	1702	1702		1665	257	114										
1989	4218	3890		2968	580	446							184	92	7	3
1990	7405	6892		5361	1178	1031							160	153	17	17
1991	11 177	10 581		8473	2015	1661							369	352	57	39
1992	17 404	16 381		12 250	2702	2525	963	936		524	42	35	553	530	79	66
1993	21 287	20 345		15 565	3730	3334	2608	2447		1271	176	149	681	597	86	71
1994	25 157	24 033		18 690	4069	3734	5510	5339		4114	759	698	1303	1112	179	144
1995	26 648	24 694		18 905	4246	3810	9820	9054		7722	1732	1579	1682	1426	323	298
1996	27 338	26 385		21 492	4818	4436	13 438	13 044		11 269	2799	2588	2900	2676	449	386
1997	32 247	30 733		24 768	5730	5060	16 573	16 376		14 275	3495	3249	5208	4958	1086	902
1998	34 929	33 670		27 436	6255	5851	18 657	18 266		15 505	3952	3701	8132	7643	1748	1567
1999	36 085	34 290		27 455	6812	5870	22 984	22 350		18 592	4702	4247	9950	9093	2198	1812
2000	31 334	29 907		24 447	6328	5447	26 712	25 794		21 067	5240	4582	11 653	10 719	2660	2245
2001	32 676	31 051		25 143	6749	5829	30 369	29 309		23 058	5924	4862	13 034	11 888	3080	2467
2002	34 953	33 849		26 854	7767	6443	34 824	33 823		25 866	6775	5486	15 887	14 759	4094	3299
2003	38 575	36 480		28 214	8336	6608	38 871	36 663		27 895	7506	5994	24 459	19 641	6205	4798
2004	41 619	39 656		29 090	8542	6709	44 698	43 628		29 946	7768	5921	30 287	24 422	7606	5538
2005	42 822	40 471		29 337	8893	6706	47 579	45 388		30 983	8019	5864	35 069	28 743	9396	6542
2006	44 778	42 248		29 440	8509	6256	52 539	49 854		32 509	7904	5401	42 171	35 804	11 798	7930
2007	53 873	52 165	7626	28 228	7416	5144	61 813	60 294	11 541	34 032	7784	5194	45 478	43 589	13 965	9257
2008	59 148	57 217	10 139	29 124	6897	4664	71 350	69 864	15 390	34 425	7017	4615	60 115	57 846	18 597	12 425
2009	63 083	60 754	11 800	28 559	6891	5046	76 790	75 340	19 046	35 167	7330	5180	73 927	71 367	23 216	16 454
2010	67 714	64 966	13 843	27 905	6556	4657	90 677	88 822	24 379	37 172	7699	5277	83 770	81 300	27 382	19 011
2011	71 422	68 651	16 202	27 284	6341	4546	10 2473	10 0518	30 773	38 098	7601	5415	95 764	92 782	31 721	22 465
2012	82 108	79 434	20 627	29 693	6703	4740	12 5229	12 2962	41 943	40 829	7947	5498	119 089	116 176	39 106	27 715
2013	89 950	87 104	25 085	30 164	6817	4776	13 4871	13 4871	49 316	41 150	8027	5630	14 1335	138 249	45 392	32 148
2014	92 269	89 397	27 624	30 414	6970	5025	14 4247	141 888	55 851	41 437	8122	5702	157 229	153 977	514 58	36 595
2015	93 614	91 079	30 498	28 858	6478	4629	155 797	153 639	63 660	41 396	8169	5761	174 740	171 495	56 888	40 611
2016	94 566	92 185	34 188	26 182	5903	4266	161 262	159 214	70 387	38 315	7324	5166	191 962	188 338	62 749	44 678
2017	91 516	89 447	36 441	22 423	5182	3731	157 709	155 758	74 200	33 297	6757	4826	198 985	195 559	67 255	48 060
2018	92 552	90 376	38 882	20 894	4755	3402	158 859	157 026	79 496	29 569	5886	4194	203 482	200 050	693 95	49 383
2019	88 074	86 334	40 561	17 341	4002	2977	154 824	153 014	83 129	24 490	4789	3433	215 203	211 758	74 911	54 188

Abbreviations: ET, embryo transfer; FET, frozen‐thawed embryo transfer; ICSI, intracytoplasmic sperm injection; IVF, in vitro fertilization.

^a^ Including gamete intrafallopian transfer.

^b^ Including split‐ICSI cycles.

^c^ Including cycles using frozen‐thawed oocyte.

**FIGURE 1:**
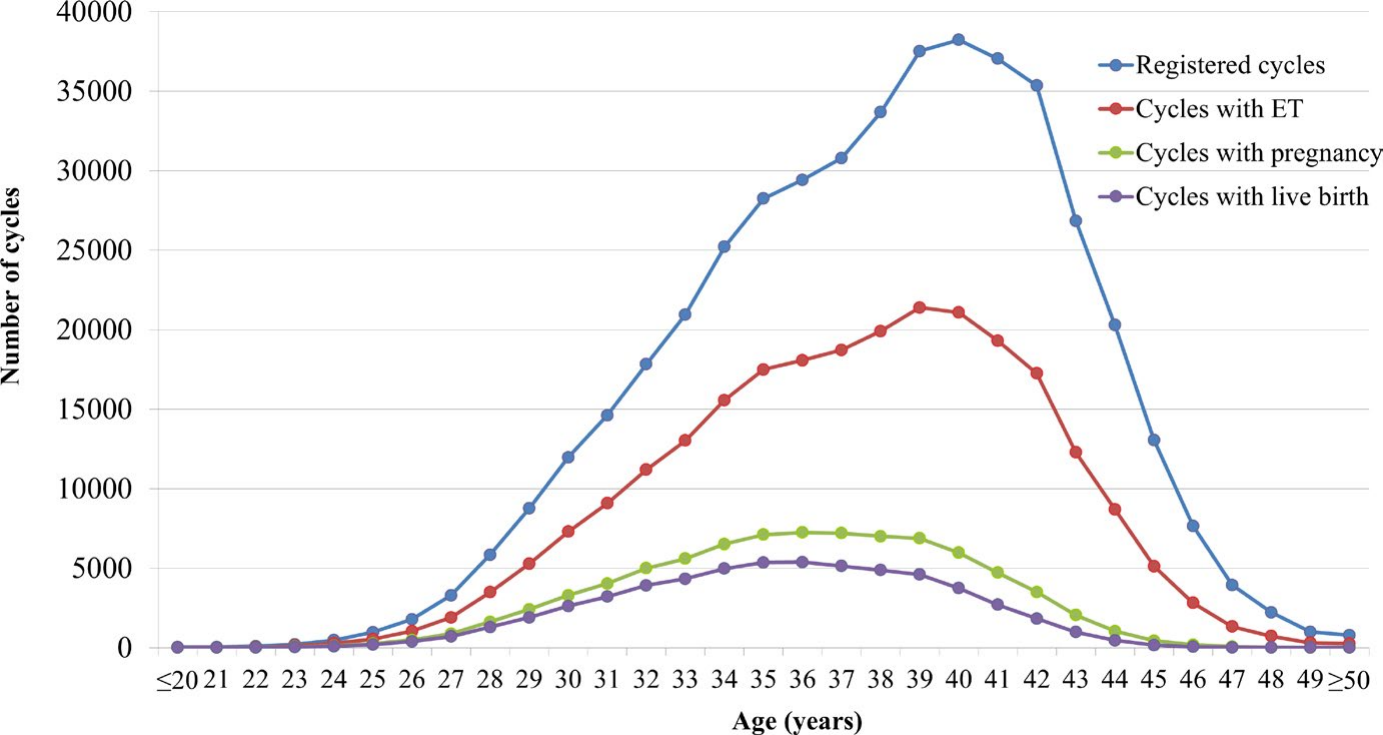
Distribution of maternal age from all registered cycles, cycles for ET, cycles leading to pregnancy, and cycles leading to live births in 2019. Adapted from the Japan Society of Obstetrics and Gynecology ART Databook 2019 (http://plaza.umin.ac.jp/~jsog‐art/2019data_202107.pdf). ET, embryo transfer

The distribution of patient age in the total number of registered cycles, cycles with ET, cycles leading to pregnancy, and cycles leading to live births is shown in Figure 1. The mean patient age for registered cycles was 37.9 years (standard deviation [SD] ± 4.7), and 40.7% of registered cycles were for women in their 40s or older; the mean age for pregnancy and live birth cycles was 35.9 years (SD ± 4.2) and 35.4 years (SD ± 4.0), respectively.

The detailed characteristics and treatment outcomes of registered fresh cycles are shown in Table 2. There were 82 908 registered IVF cycles, 28 498 split‐ICSI cycles, 124 035 ICSI cycles using ejaculated spermatozoa, 2291 ICSI cycles using testicular sperm extraction (TESE), 14 GIFT cycles, 723 cycles for oocyte freezing based on medical indications, and 4429 other cycles. Of the 239 348 cycles with oocyte retrieval, 123 690 (51.7%) were freeze‐all cycles. The pregnancy rate per ET cycle was 23.1% for IVF and 18.6% for ICSI using ejaculated spermatozoa. Single ET was performed at a rate of 82.6%, with a pregnancy rate of 21.6%. Live birth rates per ET were 16.7% for IVF, 18.4% for split‐ICSI, 12.9% for ICSI using ejaculated spermatozoa, and 7.8% for ICSI with TESE. The singleton pregnancy rate and live birth rate were both 97.3%. Of the 723 cycles for oocyte freezing based on medical indications, 717 oocyte retrievals were conducted, of which 605 cycles included the successful freezing of oocytes.

**TABLE 2:** Characteristics and treatment outcomes of registered fresh cycles in assisted reproductive technology in Japan, 2019

Variables	IVF	Split	ICSI		GIFT	Frozen oocyte	Other^a^	Total
			Ejaculated sperm	TESE				
No. of registered cycles	82 908	28 498	124 035	2291	14	723	4429	242 898
No. of egg retrievals	81 293	28 091	122 635	2288	14	717	4310	239 348
No. of fresh ET cycles	16 871	3830	20 197	463	14	0	456	418 31
No. of freeze‐all cycles	38 403	21 026	60 844	1259	0	605	1553	123 690
No. of cycles with pregnancy	3900	967	3766	56	3	0	99	8791
Pregnancy rate per ET	23.1%	25.3%	18.6%	12.1%	21.4%	‐	21.7%	21.0%
Pregnancy rate per egg retrieval	4.8%	3.4%	3.1%	2.5%	21.4%	‐	2.3%	3.7%
Pregnancy rate per egg retrieval excluding freeze‐all cycles	9.1%	13.7%	6.1%	5.4%	21.4%	‐	3.6%	7.6%
SET cycles	14 319	3284	16 216	309	1	‐	411	34 540
Pregnancy following SET cycles	3386	851	3088	47	0	‐	92	7464
Rate of SET cycles	84.9%	85.7%	80.3%	66.7%	7.1%	‐	90.1%	82.6%
Pregnancy rate following SET cycles	23.7%	25.9%	19.0%	15.2%	0.0%	‐	22.4%	21.6%
Miscarriages	901	236	1010	17	0	‐	24	2188
Miscarriage rate per pregnancy	23.1%	24.4%	26.8%	30.4%	0.0%	‐	24.2%	24.9%
Singleton pregnancies^b^	3686	931	3546	50	2	‐	94	8309
Multiple pregnancies^b^	98	23	106	2	1	‐	2	232
Twin pregnancies	97	21	105	2	0	‐	2	227
Triplet pregnancies	1	2	1	0	1	‐	0	5
Quadruplet pregnancies	0	0	0	0	0	‐	0	0
Multiple pregnancy rate	2.5%	2.4%	2.8%	3.6%	33.3%	‐	2.0%	2.6%
Live births	2821	703	2605	36	3	‐	71	6239
Live birth rate per ET	16.7%	18.4%	12.9%	7.8%	21.4%	‐	15.6%	14.9%
Total no. of neonates	2901	715	2682	36	4	‐	72	6410
Singleton live births	2742	691	2528	36	2	‐	70	6069
Twin live births	78	12	77	0	1	‐	1	169
Triplet live births	1	0	0	0	0	‐	0	1
Quadruplet live births	0	0	0	0	0	‐	0	0
Pregnancy outcomes						‐		
Ectopic pregnancies	40	10	61	1	0	‐	1	113
Heterotopic pregnancies	2	0	0	0	0	‐	0	2
Artificial abortions	24	3	17	0	0	‐	0	44
Still births	12	2	13	0	0	‐	0	27
Fetal reductions	1	1	1	0	1	‐	0	4
Cycles with unknown pregnancy outcomes	42	11	42	1	0	‐	2	98

Abbreviations: ET, embryo transfer; GIFT, gamete intrafallopian transfer; ICSI, intracytoplasmic sperm injection; in vitro fertilization; IVF; SET, single embryo transfer; TESE, testicular sperm extraction; ZIFT, zygote intrafallopian transfer.

^a^ Others include ZIFT.

^b^ Singleton, twin, triplet, and quadruplet pregnancies were defined according to the number of gestational sacs in utero.

The characteristics and treatment outcomes of FET cycles are shown in Table 3. There was a total of 214 938 registered cycles in 2019, of which FET was performed in 211 597, leading to 74 882 pregnancies (pregnancy rate per FET = 35.4%). The miscarriage rate per pregnancy was 25.4%, resulting in a 24.9% live birth rate per ET. Single ET was performed at a rate of 85.1%, and the singleton pregnancy and live birth rates were 97.1% and 97.3%, respectively.

**TABLE 3:** Characteristics and treatment outcomes of frozen cycles in assisted reproductive technology in Japan, 2019

Variables	FET	Other^a^	Total
No. of registered cycles	213 882	1056	214 938
No. of FET	210 656	941	211 597
No. of cycles with pregnancy	74 595	287	74 882
Pregnancy rate per FET	35.4%	30.5%	35.4%
SET cycles	179 335	790	180 125
Pregnancy following SET cycles	65 249	256	65 505
Rate of SET cycles	85.1%	84.0%	85.1%
Pregnancy rate following SET cycles	36.4%	32.4%	36.4%
Miscarriages	18 957	68	19 025
Miscarriage rate per pregnancy	25.4%	23.7%	25.4%
Singleton pregnancies^b^	71 088	278	71 366
Multiple pregnancies^b^	2160	4	2164
Twin pregnancies	2118	4	2122
Triplet pregnancies	40	0	40
Quadruplet pregnancies	2	0	2
Multiple pregnancy rate	2.9%	1.4%	2.9%
Live births	52 513	214	52 727
Live birth rate per FET	24.9%	22.7%	24.9%
Total no. of neonates	53 949	219	54 168
Singleton live births	51 084	209	51 293
Twin live births	1419	5	1424
Triplet live births	9	0	9
Quadruplet live births	0	0	0
Pregnancy outcomes			
Ectopic pregnancies	398	0	398
Intrauterine pregnancies coexisting with ectopic pregnancy	10	0	10
Artificial abortions	363	2	365
Still births	196	0	196
Fetal reductions	18	0	18
Cycles with unknown pregnancy outcomes	1655	2	1657

Abbreviations: FET, frozen‐thawed embryo transfer; SET, single embryo transfer.

^a^ Including cycles using frozen‐thawed oocytes.

^b^ Singleton, twin, triplet, and quadruplet pregnancies were defined according to the number of gestational sacs in utero.

**FIGURE 2:**
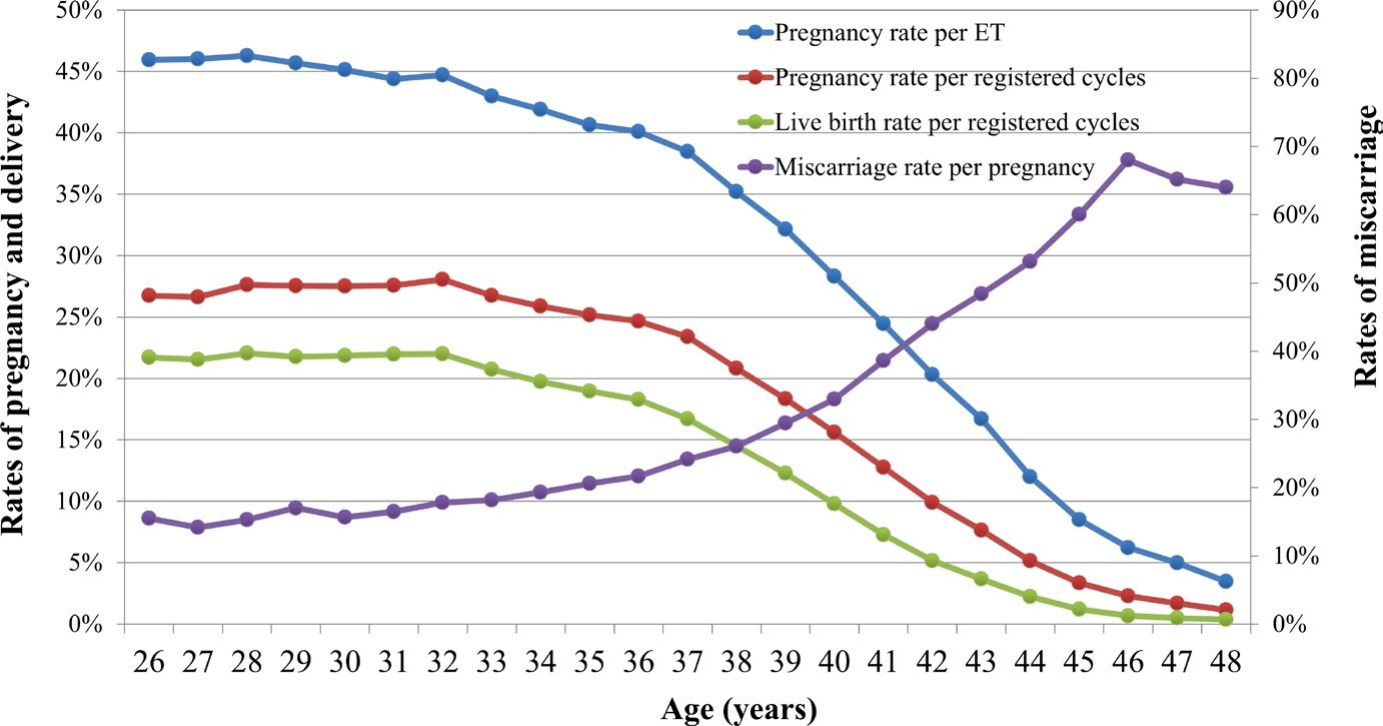
Pregnancy, live birth, and miscarriage rates according to patient age in all registered cycles in 2019. Adapted from the Japan Society of Obstetrics and Gynecology ART Databook 2019 (http://plaza.umin.ac.jp/~jsog‐art/2019data_202107.pdf). ET, embryo transfer

Treatment outcomes of registered cycles, including pregnancy, miscarriage, live birth, and multiple pregnancy rate, according to maternal age, are shown in Table 4. The pregnancy, live birth, and miscarriage rate according to maternal age are shown in Figure 2. The pregnancy rate per ET exceeded 40% up to a maternal age of 36 years; this rate gradually fell below 30% for women aged over 40 years and below 10% for women aged over 45 years. The miscarriage rate was below 20% for women aged less than 35 years, but gradually increased to 32.9% and 48.4% for those aged 40 and 43 years, respectively. The live birth rate per registered cycle was around 20% in women up to 33 years of age and decreased to 9.8% and 3.6% for women aged 40 and 43 years, respectively. The multiple pregnancy rate varied between 2% and 3% for most age groups (Table 4).

**TABLE 4:** Treatment outcomes of registered cycles according to patient age in Japan, 2019

Age (years)	No. of registered cycles	No. of ET cycles	Pregnancy	Multiple pregnancies^a^	Miscarriage	Live birth	Pregnancy rate per ET	Pregnancy rate per registered cycles	Live birth rate per registered cycles	Miscarriage rate per pregnancy	Multiple pregnancy rate^a^
≤20	43	6	4	0	0	4	66.7%	9.3%	9.3%	0.0%	0.0%
21	38	16	9	1	3	6	56.3%	23.7%	15.8%	33.3%	0.0%
22	83	35	15	0	2	11	42.9%	18.1%	13.3%	13.3%	9.5%
23	210	117	53	4	7	42	45.3%	25.2%	20.0%	13.2%	5.7%
24	473	270	111	1	19	87	41.1%	23.5%	18.4%	17.1%	1.0%
25	974	538	247	10	39	196	45.9%	25.4%	20.1%	15.8%	0.0%
26	1788	1041	478	13	74	388	45.9%	26.7%	21.7%	15.5%	4.0%
27	3288	1902	875	22	124	708	46.0%	26.6%	21.5%	14.2%	1.7%
28	5844	3488	1614	41	247	1289	46.3%	27.6%	22.1%	15.3%	2.2%
29	8751	5278	2411	58	410	1904	45.7%	27.6%	21.8%	17.0%	3.4%
30	11 974	7298	3293	84	515	2616	45.1%	27.5%	21.8%	15.6%	3.0%
31	14 616	9076	4028	108	664	3208	44.4%	27.6%	21.9%	16.5%	2.6%
32	17 830	11 187	5000	143	891	3919	44.7%	28.0%	22.0%	17.8%	2.6%
33	20 944	13 027	5598	129	1017	4341	43.0%	26.7%	20.7%	18.2%	2.6%
34	25 210	15 566	6522	188	1259	4975	41.9%	25.9%	19.7%	19.3%	2.9%
35	28 249	17 489	7105	226	1464	5356	40.6%	25.2%	19.0%	20.6%	3.2%
36	29 414	18 078	7249	221	1571	5377	40.1%	24.6%	18.3%	21.7%	3.8%
37	30 787	18 727	7201	229	1739	5139	38.5%	23.4%	16.7%	24.1%	3.0%
38	33 672	19 907	7006	232	1825	4880	35.2%	20.8%	14.5%	26.0%	3.3%
39	37 516	21 394	6879	217	2025	4605	32.2%	18.3%	12.3%	29.4%	3.0%
40	38 221	21 075	5967	173	1966	3740	28.3%	15.6%	9.8%	32.9%	2.8%
41	37 040	19 299	4719	133	1821	2694	24.5%	12.7%	7.3%	38.6%	2.8%
42	35 349	17 243	3501	80	1541	1823	20.3%	9.9%	5.2%	44.0%	2.8%
43	26 845	12 284	2050	47	992	979	16.7%	7.6%	3.6%	48.4%	3.0%
44	20 291	8697	1043	20	554	453	12.0%	5.1%	2.2%	53.1%	3.1%
45	13 066	5125	435	8	261	157	8.5%	3.3%	1.2%	60.0%	1.2%
46	7651	2816	175	5	119	51	6.2%	2.3%	0.7%	68.0%	1.4%
47	3938	1327	66	0	43	19	5.0%	1.7%	0.5%	65.2%	1.9%
48	2217	723	25	1	16	8	3.5%	1.1%	0.4%	64.0%	6.3%
49	991	313	11	2	7	4	3.5%	1.1%	0.4%	63.6%	0.0%
≥50	788	251	12	0	5	7	4.8%	1.5%	0.9%	41.7%	0.0%

Abbreviation: ET, embryo transfer.

^a^ Multiple pregnancies were defined according to the number of gestational sacs in utero.

The treatment outcomes of cycles using frozen‐thawed oocytes are shown in Table 5. There were 161 FET cycles using frozen‐thawed oocytes in 2019, of which 29 cycles resulted in a pregnancy (pregnancy rate per FET = 18.0%). The miscarriage rate per pregnancy was 24.1%, resulting in a 12.4% live birth rate per ET.

**TABLE 5:** Treatment outcomes of embryo transfers using frozen‐thawed oocytes in assisted reproductive technology in Japan, 2019

Variables	Embryo transfers using frozen‐thawed oocyte
No. of registered cycles	265
No. of ET	161
No. of cycles with pregnancy	29
Pregnancy rate per ET	18.0%
SET cycles	117
Pregnancy following SET cycles	24
Rate of SET cycles	72.7%
Pregnancy rate following SET cycles	20.5%
Miscarriages	7
Miscarriage rate per pregnancy	24.1%
Singleton pregnancies^a^	27
Multiple pregnancies^a^	0
Twin pregnancies	0
Triplet pregnancies	0
Quadruplet pregnancies	0
Multiple pregnancy rate	0.0%
Live births	20
Live birth rate per ET	12.4%
Total number of neonates	20
Singleton live births	20
Twin live births	0
Triplet live births	0
Quadruplet live births	0
Pregnancy outcomes	
Ectopic pregnancies	0
Intrauterine pregnancies coexisting with ectopic pregnancy	0
Artificial abortions	0
Still births	0
Fetal reductions	0
Cycles with unknown pregnancy outcomes	0

Abbreviations: ET, embryo transfer; SET, single embryo transfer.

^a^ Singleton, twin, triplet, and quadruplet pregnancies were defined according to the number of gestational sacs in utero.

## 4 DISCUSSION

In this descriptive study, we evaluated and reported on the main characteristics and treatment outcomes of ART cycles administered at 595 Japanese ART facilities during 2019. Moreover, we compared the number and types of ART treatments registered in 2019, including their outcomes, with previous years. The Japanese ART registry system is managed by the JSOG and is crucial for understanding and increasing the low fertility rate in Japan, as well as for evaluating the safety of ART techniques and understanding the latest trends regarding their implementation in Japan.

In 2019, 458 101 ART cycles were registered by the JSOG in Japan, and they resulted in the birth of 60 598 neonates. Both of these are higher than the reported numbers for 2018.^3^ Compared with findings in 2018,^3^ the number of fresh cycles, including IVF and ICSI, decreased in 2019. Freeze‐all cycles were the predominant reported cycle type; in 2019, 51.7% of cycles with oocyte retrieval for IVF and ICSI combined were freeze‐all cycles, whereas the percentage attributable to freeze‐all cycles in 2018 was 47.8%. Therefore, the number of freeze‐all cycles in 2019 was higher than the previous year, a trend that has been observed for the past 5 years.^3^, ^4^, ^5^, ^6^, ^7^ Moreover, the number of registered IVF‐ET and ICSI cycles that resulted in a neonate birth in 2019 (2974 and 3433, respectively) was lower compared with the reported numbers of the previous year.^3^ There was a 5.8% increase in FET cycles in 2019 compared with 2018, including a total of 215 203 cycles that resulted in 74 911 pregnancies and 54 188 neonates. Conversely, the single ET rate was 82.6% for fresh cycles and 85.1% for FET cycles, which continues the previously reported gradual increase in the use of these techniques. For fresh cycles, the singleton pregnancy and live birth rates each reached 97.3%, and for frozen cycles, the singleton pregnancy and live birth rates reached 97.1% and 97.3%, respectively, which are also similar values to those reported in 2018.

We analyzed the distribution of cycle type and outcome based on maternal age. The mean patient age for registered cycles in 2019 was 37.9 years (SD ± 4.7). Of all registered cycles in 2019, 40.7% were undertaken by women aged ≥40 years, which is similar to the percentage of women aged ≥40 years undergoing ART cycles in 2018 (41.8%). The rate of pregnancy was above 40% for women aged 36 years or younger undergoing ART; in women aged >36 years, the pregnancy rate with ART declined progressively with increasing age. Conversely, the rate of miscarriage remained <20% in women under 34 years of age undergoing ART, and the rate gradually increased with increasing age in women older than 34 years. However, the data indicated a miscarriage rate of 33.3% in women aged 21 years, which deviates from the abovementioned trend. Because the number of women receiving ART in this age group was very low (in 2019, 38 cycles were registered and 9 pregnancies were recorded), this rate is likely a result of random variation. In addition, live birth rates per cycle were approximately 20% in women aged 33 years or younger, and this gradually decreased with increasing age in women aged >33 years, down to 3.6% in patients who were 43 years old.

Over the past 40 years in Japan, the fertility rate has continuously declined.^2^ This is a significant issue that has become a priority for the government in recent decades. Although several policies and countermeasures have been implemented by the government in Japan, these changes are not necessarily beneficial for all people. For instance, some policies have included increased assistance for childcare and additional support for a work–life balance; however, these are mostly targeted at two‐income households and are not available for single individuals who are not married or not in a relationship. Therefore, despite these measures, the fertility rate is still decreasing. Compared with a total fertility rate of 1.42 births per woman in 2018,^8^ the fertility rate in 2019 was 1.36 births per woman.^9^

In January 2021, several incentives and subsidies were put in place as part of a support program for couples receiving ART. This policy change included the removal of income limitations (previously, couples had to have an annual income of under 7 300 000 JPY to be eligible for support), an increase in the subsidy available per cycle (from 300 000 JPY for the first cycle and 150 000 JPY for each subsequent cycle to 300 000 JPY for each cycle), and an increase in the number of cycles available per woman/couple (from six cycles in total to six cycles per child for women aged under 40 years and three cycles per child for women aged 40–43 years). The existing age limitation for eligibility (age <43 years at the time of starting treatment) remained in place.^10^ These subsidies are covered in part by the state and the implementing entities, such as the prefecture, ordinance‐designated city, or core city, and the project‐related procedures and treatments are carried out at designated clinics.

The Japanese government is planning to further expand ART provision to provide universal insurance coverage to couples with fertility issues who wish to undertake ART. The full details of the specific procedures, costs, and coverages are still under discussion at the Central Social Insurance Medical Council.^11^, ^12^ The Japan Society for Reproductive Medicine has prepared a guideline regarding the ART insurance coverage changes, which will be published in November 2021 in advance of the policy rollout in 2022. The potential impact of the coverage changes on the number of treatment cycles and the number of infants born from ART, as well as the health insurance costs, is unknown. Therefore, it is not clear whether the coverage will considerably increase the accessibility of ART. Government‐assisted health coverage programs for ART are currently available for eligible candidates in Israel, Canada, Denmark, and the UK^13^; although outcome data from these programs are sparse, a recent report of the 5‐year government health coverage experience in Quebec compared with a non‐insured period showed a remarkable increase in the number of ART cycles performed during the insured period, despite increased cycle cancellation rates.^14^ This shows that government funding for ART and/or subsidized ARTs may make fertility treatments more accessible to the public and will therefore have a positive overall socioeconomic impact. Whether the implementation of these programs will influence data collection or other aspects of the ART registry by the JSOG remains undetermined.

The JSOG recently completed a pilot study that investigated the effectiveness of preimplantation genetic testing for aneuploidy (PGT‐A) for improving the live birth rate and reducing miscarriage rates in specific populations. The specific population in this study included patients experiencing recurrent pregnancy loss (RPL) caused by an abnormal embryonic karyotype and patients experiencing recurrent implantation failure (RIF) or ART failure. The results of the pilot study showed that PGT‐A did not improve the number of live births or decrease the number of miscarriages in the RPL or RIF groups; however, it did improve the live birth rate per ET in both groups.^15^ Thus, PGT‐A reduced the number of ETs required to achieve a similar number of live births compared with those not undergoing PGT‐A. The main study following this pilot is currently underway as a multicenter prospective study. Although the main results will not be available until 2022, the preliminary findings of the pilot study suggest that the forthcoming results may support the use of this screening method for detecting euploid embryos in specific populations in Japan. Although several studies and systematic reviews have been conducted on the effectiveness of PGT‐A for this purpose,^16^, ^17^, ^18^, ^19^ the results remain controversial. However, from a clinical standpoint, PGT‐A may be of particular benefit for women in their mid‐ or late‐30s to facilitate single ET and reduce RPL, RIF, and ART failure, and shorten time to pregnancy,^20^ all of which are problems faced by this age group.

In the present study, we found that FET increased in frequency in 2019 compared with 2018,^3^ which is consistent with the trend observed for FET cycles over the past 5 years in Japan. Interestingly, the decreasing trends in Japan regarding the use of fresh cycles are different to those observed in other Asia‐Pacific countries. For instance, in New Zealand and Australia, the trend indicates an increase in fresh cycles and a reduction in FET cycles in the last 5 years.^21^ Therefore, it is possible that the total number of women receiving ART may be decreasing in Japan. Although fresh ET continues to be the major type of ART in Japan, there are several concerns regarding controlled ovarian stimulation (COS) for the endometrium,^22^ which may result in lower pregnancy rates and poorer obstetric and perinatal outcomes compared with FET. The freeze‐all strategy is becoming more widely used in Japan because of several key factors, including an improvement in cryopreservation techniques, the advantages of delayed FET, fewer complications related to ovarian stimulation (ie, COS and ovarian hyperstimulation syndrome), development of new ovarian stimulation protocols such as progestin‐primed ovarian stimulation, and reports of significantly higher implantation, clinical pregnancy, and live birth rates with freeze‐all versus fresh cycles.^23^, ^24^ However, several studies have reported that the advantages of the freeze‐all strategy over fresh ET are highly dependent on the number of oocytes retrieved (threshold of >15 oocytes) and that in procedures with oocyte counts below this threshold, these advantages are insignificant.^23^, ^24^, ^25^ Moreover, several researchers argue that the freeze‐all strategy should be reserved for specific patient populations, such as those with a risk of ovarian hyperstimulation syndrome and those with supra‐physiologic hormonal levels during the follicular phase of COS.^26^ Moreover, it remains unclear whether this strategy is beneficial for patients with regular menstrual cycles.^26^, ^27^, ^28^, ^29^

This study has several strengths and limitations. The main strength of the Japanese ART registry system is that the registration of ART cases is mandatory in the designated ART facilities in Japan and the compliance rate is very high. Additionally, the reporting and recording of data are standardized, meaning cycle‐specific information is collected in the same way nationwide; thus, the risk of reporting bias is low. However, the collection of patient background data is not standardized or mandatory; therefore, the large amount of patient background data that was missing because of low reporting is a major limitation of this study. For instance, data on body mass index, the number of previous pregnancies, and parity were unavailable for some patients. Therefore, to improve the accuracy of analyses on these data, the reporting of such variables should be encouraged. Furthermore, other background information, such as the presence of relevant patient conditions (eg, polycystic ovarian syndrome and anovulation) and treatment history, should be collected, as these may have a significant impact on the outcome of ART. These types of information will be included in the registries from 2022. Because the collection of data for the registry was carried out per cycle, we were not able to distinguish whether a person received multiple treatment cycles. Thus, the information required to calculate the cumulative live birth rate or pregnancy rate per patient receiving ART treatment is currently unavailable, and this is a significant limitation of the registry.

In conclusion, the present analysis of the ART registry showed that the overall number of ART cycles and neonates born increased in 2019 compared with 2018, and this increase was largely because of an increased number of freeze‐all cycles in 2019 (a 5.8% increase from 2018). The rate of single ETs, both fresh and frozen, also increased in 2019, but the increase was most striking for FET cycles. For both fresh and FET cycles, the rates of singleton pregnancies and live births were over 97%. In summary, the trends for ART use and outcomes in Japan in 2019 were similar to those of the previous year. It will be interesting to compare these results with the forthcoming results after the implementation of the Japanese ART subsidy programs and the government health coverage for ART.

## CONFLICT OF INTEREST

There is no conflict of interest regarding the publication of this study.

## HUMAN RIGHTS STATEMENTS AND INFORMED CONSENT

All procedures were performed in accordance with the ethical standards of the relevant committees on human experimentation (institutional and national) and the Helsinki Declaration of 1964 and its later amendments.

## ANIMAL RIGHTS

This report does not contain any studies performed by any of the authors that included animal participants.

## APPROVAL BY ETHICS COMMITTEE

Not applicable.

## CLINICAL TRIAL REGISTRY

Not applicable.
